# An integrative pharmacological approach to radio telemetry and blood sampling in pharmaceutical drug discovery and safety assessment

**DOI:** 10.1186/1475-925X-10-5

**Published:** 2011-01-18

**Authors:** Dennis C Litwin, David J Lengel, Harriet W Kamendi, Russell A Bialecki

**Affiliations:** 1 Safety Assessment US, AstraZeneca R&D Wilmington, DE 19850, USA

## Abstract

**Background:**

A successful integration of the automated blood sampling (ABS) and telemetry (ABST) system is described. The new ABST system facilitates concomitant collection of physiological variables with blood and urine samples for determination of drug concentrations and other biochemical measures in the same rat without handling artifact.

**Method:**

Integration was achieved by designing a 13 inch circular receiving antenna that operates as a plug-in replacement for the existing pair of DSI's orthogonal antennas which is compatible with the rotating cage and open floor design of the BASi Culex^® ^ABS system. The circular receiving antenna's electrical configuration consists of a pair of electrically orthogonal half-toroids that reinforce reception of a dipole transmitter operating within the coil's interior while reducing both external noise pickup and interference from other adjacent dipole transmitters.

**Results:**

For validation, measured baclofen concentration (ABST vs. satellite (μM): 69.6 ± 23.8 vs. 76.6 ± 19.5, p = NS) and mean arterial pressure (ABST vs. traditional DSI telemetry (mm Hg): 150 ± 5 vs.147 ± 4, p = NS) variables were quantitatively and qualitatively similar between rats housed in the ABST system and traditional home cage approaches.

**Conclusion:**

The ABST system offers unique advantages over traditional between-group study paradigms that include improved data quality and significantly reduced animal use. The superior within-group model facilitates assessment of multiple physiological and biochemical responses to test compounds in the same animal. The ABST also provides opportunities to evaluate temporal relations between parameters and to investigate anomalous outlier events because drug concentrations, physiological and biochemical measures for each animal are available for comparisons.

## Background

Integrative (i.e., *in vivo*) pharmacology [1,2] remains an essential part of drug discovery. Regulatory authorities require pharmaceutical industries to demonstrate *in vivo *efficacy and safety properties of new therapeutic entities using measured drug concentrations and effects in various tissues [3]. Ideal responses associated with drug administration are best obtained by measuring relevant biochemical endpoints and physiological variables such as blood pressure, heart rate and core body temperature as well as biopotentials like electroencephalogram (EEG) or, electrocardiogram (ECG) while comparing them to drug exposure in the same animal. Methods in use today are commonly restricted to multiple and separate studies conducted by different scientific disciplines to acquire this information due to lack of instrumentation that can accurately perform all measurements simultaneously.

PhysioTel Multiplus series radio telemetry transmitters (Data Sciences International, St. Paul, MN) for use in laboratory animals allow assessment of multiple physiological variables simultaneously in one animal. While the advent of this technology improved acquisition of physiological data in conscious freely moving and undisturbed animals, data collection is often times confounded by noisy signals resulting from "the animal handling artifact" during both drug delivery and procurement of blood samples. The common "work around" for the handling artifact involves using a separate (i.e., satellite) group of animals to obtain blood samples while recording physiological changes in the first group of animals. Satellite animals are effective in establishing average limits for drug exposure, especially if pharmacokinetic variables such as bioavailability, maximum concentration, volume of distribution and clearance remain constant. However, pharmacokinetic properties of drugs differ between animals due to individual variability. In addition, it is often difficult to compare concentration-response relationships in different groups of animals due to variations in experimental methodology and covariates. By comparison, collecting all experimental variables from the same animal helps rectify these disparities. Hence the objective of this work was to develop an experimental model for acquisition of multiple physiologic variables *in vivo *using radio telemetry concomitant with automated blood sampling.

## Methods

### Reasons for integration of the BASi Culex^® ^and DSI radio telemetry

Efforts to reduce the number of animals used in the drug discovery and safety assessment process while maintaining a valid integrative pharmacological approach are presented here. Success of this effort highlights the importance of an effective working collaboration among a diverse group of scientists, engineers, technicians and instrument companies throughout the integration process.

The system described herein represents an integration of two 'stand-alone' commercially available instruments, the PhysioTel Multiplus series transmitters: radio telemetry system for rodents (DSI, St. Paul, MN) and the Culex^® ^automated blood sampling (ABS) system (BAS Inc, West Lafayette, IN). The highly automated Culex^® ^ABS system allows for unattended blood sample collection via an in-dwelling arterial or venous catheter. Samples are collected at user-programmed times and volumes. The Culex^® ^system can accommodate awake and freely moving animals such as the rat. The system is designed so that contiguous tubing, devoid of liquid swivels and commutators, is used to cannulate the animal. Twisting and/or pinching of sampling lines is avoided by housing the animal on a turntable that rotates to counter the direction of the animal's movement. The turntable supports the circumference of a metabolism cage floor that enables collection of urine and feces. Blood and urine samples are maintained at cold temperatures until the operator removes them. More information on this system can be found at: http://www.basinc.com/products/culex/index.html.

The second stand-alone system provides a continuous stream of physiological data from an implanted transmitter and its analog sensors for blood pressure, EEG or ECG, core body temperature and activity (DSI Physio Tel Multiplus series transmitters: model TL11M2-C50-PXT). The external portion includes the Physio Tel receiver (DSI model RPC-1) as well as software for data acquisition and analysis (http://www.datasci.com/products/implantable_telemetry/transmitters.asp). When used as originally designed, a single animal housed in a home cage is placed on the DSI PhysioTel receiver. The PhysioTel receiver is intended to be in close proximity to its transmitter. Unfortunately, this configuration is incompatible with the Culex^® ^ABS system due to the existence of the urine and feces collection unit that prevents conventional placement of the receiving antenna.

### Integration of the two commercial systems

The first attempt at integrating the two systems involved placing the Physio Tel receiver at a 90-degree angle next to the cage. The receiver was placed at an increased distance from the cage to provide clearance for food and water attachments that rotated along with the cage. With this configuration, data was either lost completely or greatly diminished. Rotation of the receiver to a 45-degree angle improved transmitter-receiver signal sharing, but still suffered from significant data loss when the animal was on the opposite side of the cage. This loss of data was accompanied by increased background noise level resulting from automatic gain circuit in the Physio Tel receiver compensating for the weaker transmitter signal. The antenna orientation problem could be partially resolved by adding a second Physio Tel receiver placed across from the first. The use of two channels and two receivers for each cage allowed detection of the desired signal with a tolerable level of signal drop-outs on one or the other of the two channels. Additional complications concerning the physical placement of the receivers included the fact that two receivers per Culex^® ^cage prohibited use of half the available Culex^® ^chambers and increased the per-cage expense.

The second attempt at integration involved the design of a simple helical coil receiving antenna that would place the transmitter in its interior. Examination of the operating Culex^® ^system showed that there is only a small space near the bottom of the rotating cage available for antenna placement with a minor complication involving the need to open or close the cage door. A ring of unobstructed space with a 5.5" radius that completely surrounds the cage was obtained by cutting approximately ½" from the bottom of the cage door.

**Figure 1:**
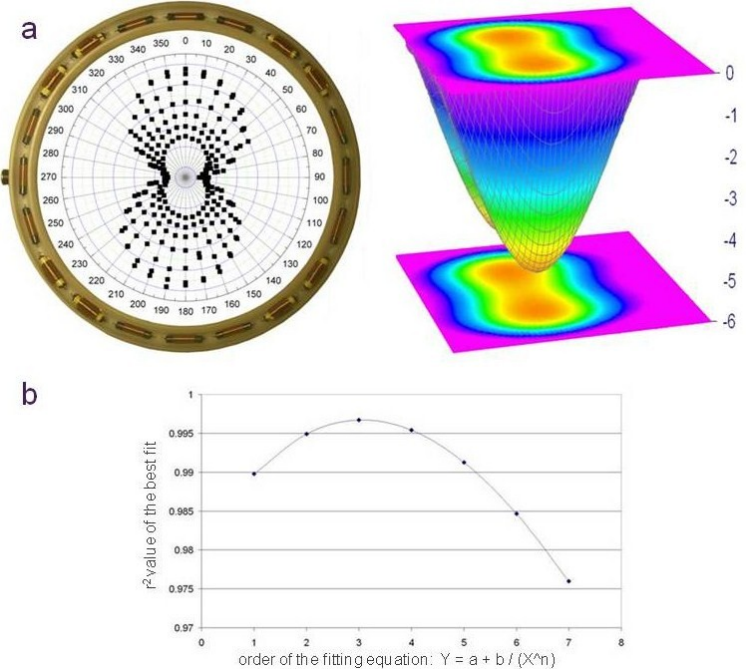
**Transmitter field-strength pattern and surface curve-fit.**  (a) Transmitter field-strength pattern and a surface curve-fit to the calculated dipole transmitter pattern for L/λ = 0.2. (b) Signal Fall-off indicates the primarily cubic nature of the observed fall-off in voltage signal strength vs. receiver to antenna separation. Equations of the form Y = a + b/(X^n) n = 1, 7 were fitted to the data with the "best fit" for each order being reported as its r-square value.

Antenna design required knowledge of what was being transmitted and also what was being received. The transmitter was found to be very directional, exhibiting a dipole-like radiation pattern. Constant field-strength patterns were taken at increasing 1" distances in the vertical plane, starting from the coplanar pattern which is presented as the largest two lobes in Figure 1a. All points represent identical measures of field strength. Measurements were taken in the plane of the transmitter and repeated at increasing one inch elevations above the transmitter to a maximum of six inches above the transmitter that remained on the cage floor. The pattern for each elevation can be seen in Figure 1a, as "0" for the coplanar measures and "6" as the greatest out of plane measure. The measurement at "6" corresponds to a transmitter being six inches above the cage floor. It can be seen in Figure 1b that this is a reasonable pattern for a simple dipole antenna. Animations, courtesy of Dr. Dan Russell, Kettering University and Yannopoulou and Zimourtopoulos, Wolfram Demonstrations Project [4-6] elegantly demonstrate why two lobes are observed in the intensity vs. direction diagram. Although the animation is intended for sound waves, the principles are identical. DSI engineers indicated that the antenna-to-receiver detected voltage level shows an inverse cubic fall-off vs. separation distance. This was tested by a curve fit of the signal strength data measured at the various transmitter-to-antenna separations (Figure 1c). Near-field conditions [7] exist when the distance between the receiver and transmitter is less than the wavelength of the detected signal. Fitting the data to equations of the form y = a + b/X^n confirmed the best fit to a cubic relationship (Figure 1c) providing further confirmation that our measurements were in agreement with DSI. The rapid signal fall-off and the transmitter's directionality complicated integration of the two systems, reinforcing the need for an efficient receiving antenna. In addition, the new antenna system needed to be electrically equivalent to the original pair of orthogonal ferrite antennas so that the user could continue to tune the receiver's front-end electronics to the antenna without the need to alter existing receiver circuitry.

Determining an optimal antenna configuration is facilitated by knowing detailed information about DSI's Physio Tel receiver circuitry. The issue of reverse engineering deserves mention as it is often encountered while integrating existing commercial systems. It is both unethical and illegal to reverse engineer another party's intellectual property without prior agreement. Once a mutual non-disclosure agreement protecting DSI's intellectual property rights was in place, we were granted full access to their engineers and relevant sections of their receiver.

A 13" inch helical receiving antenna was constructed using Wheeler's formulas [8] to the target inductance of one of DSI's ferrite core antenna coils. The inductance was subsequently refined by direct measurements using a Quad Tech model 1920 precision LCR meter. Initial trials confirmed that an equivalent antenna would be able to work with the existing circuitry and be effective in detecting the transmitter in a limited range of orientation. The test was to see if a transmitter located within the center of a simple helical coil would be sensed in all orientations; it was not. As constructed (Figure 1a), the test antenna required that the transmitter be vertical for optimal coupling. Vertical orientation of the transmitter is only achieved when the animal is rearing due to the transmitter's orientation in the abdomen. Thus, a toroidal receiving coil antenna configuration was clearly indicated by the first test. As DSI's receiver circuit presents a pair of antenna connections, a pair of toroidal antennas was also indicated. Therefore, a pair of electrically orthogonal half-toroids became the design target.

**Figure 2:**
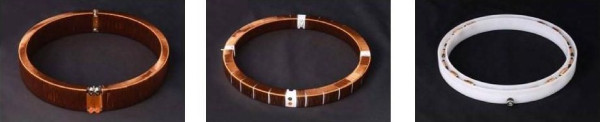
**Antenna Design Progression**. Design progression from internal radial windings (a) to outer axial windings forming two half-toroids. (b) Quarter coils with opposing pairs connected (connections not shown). The final design (c) is an approximation to the quarter coils in (b) using commercially available inductors.

Initial observations confirmed that proper tuning of the receiver circuit, transmitter orientation, and proximity of antenna to the transmitter are all fundamental concerns with this type of radio telemetry system. These considerations are well described [9]. Briefly, inductive antennae that are physically smaller than the radian wavelength (1/2π * wavelength) act as simple lumped inductances. A small vertical coil radiates a directional pattern that fills about 2/3 of the solid angle of a sphere with a radiation pattern similar to a horizontal doughnut. And finally, the efficient use of a small antenna requires accurate tuning to the frequency of interest. A second test antenna consisting of four quarter coils was tested. Optimal results were obtained when two opposing sets of quarter coils were cross-connected to form two pairs of orthogonal coils (Figure 2). This is due to the constructive addition of the two out of phase lobes that the transmitter generates. An additional benefit realized from this antenna configuration is that signals originating from outside of the array combine destructively. Thus, the coil configuration effectively couples with the transmitter while simultaneously decreasing common-mode background noise pick-up. This effect is seen to be important as four of these Physio Tel receiver assemblies are placed in close proximity to each other on the Culex^® ^station. The Physio-Tel receivers' automatic gain circuitry is extremely good at following a fading signal so that temporary pickup of a nearby transmitter would be expected; however, the noise-cancelling antenna configuration prevents this. Consequently, no shielding between antennas is required.

**Figure 3:**
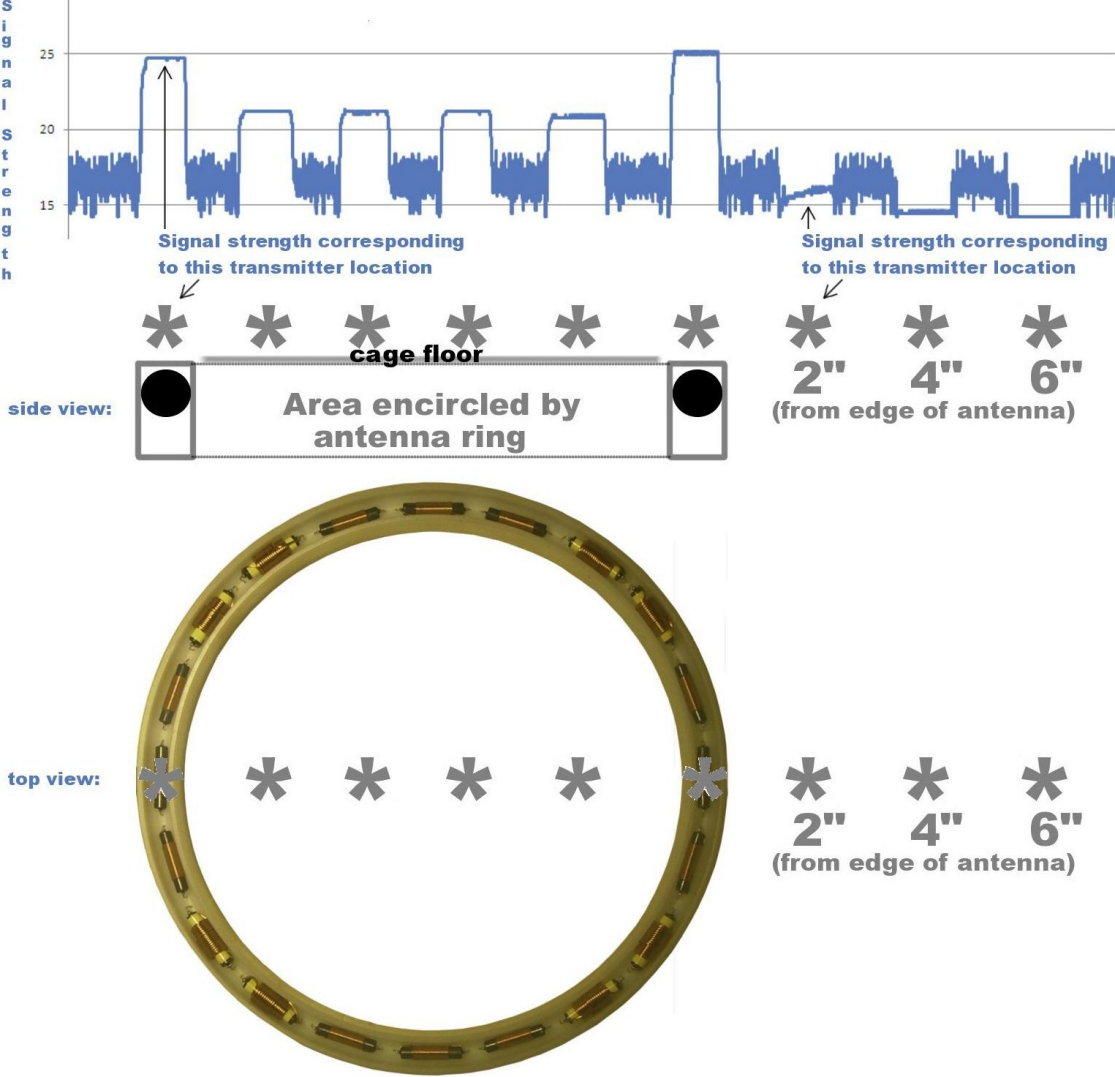
Signal strength captured across the interior of the 13" diameter and at increasing distance from outside the perimeter of the Circular receiver.

The final modification to the receiving coil assembly was predicated by ease of manufacture and the need to keep the receiving antennas clean in the laboratory setting. Winding coils around the plastic forms was tedious and the exposed wire antennas were difficult to keep clean. As a solution to this problem, each quarter coil was replaced by a set of commercially available ferrite core inductors that were selected to present the required final inductance values. These inductors were potted in a hollowed plastic ring and filled with silicone elastomer (Figure 2). A connector was added to each of the receiver bases that allowed a quick disconnection of the antenna from existing DSI circuitry (Figure 2). The original axially wound coil was added back to one of these assemblies to determine if the quarter coils and the simple circular antenna would interfere. There was no interference observed and the optional 3^rd ^axis antenna provides the possibility for measuring either rearing behavior or providing better coupling when the animal is vertically exploring the enclosure. However, since the PhysioTel receiver only offers two inputs, the third antenna is unused. Subsequent signal strength measurements of the transmitter indicated that it was detected uniformly within the antenna ring's interior area and not detected at all in the antenna exterior (Figure 3).

### Validating the ABST telemetry model

All animal procedures were performed with the approval of the internal Institutional Animal Care and Use Committee in accordance with the recommendations of the panel on euthanasia of the American Veterinary Medical Association and the National Institutes of Health publication, "Guide for the Care and Use of Laboratory Animals [10]."

After the ABST system was engineered and constructed, it required validation. For this, physiological responses to baclofen (10 mg/Kg, p.o.) or vehicle (0.9% saline solution p.o.) were recorded and compared using animals either housed in the ABST system or in home cages resting atop DSI Physio Tel receivers in their traditional orientation. This study also demonstrated the ease of using the ABST system versus home caged animals.

**Figure 4:**
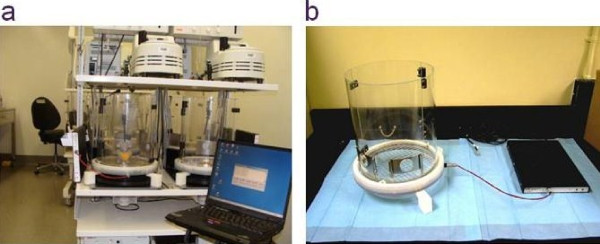
**ABST System**. The picture of the Automated Blood Sampler represents (a) the BASi Culex^® ^cage with the reengineered DSI Physio Tel receiver (b) a close up picture of the Culex^® ^ABS cage, circular antenna and the DSI Physio Tel receiver.

Twenty Han Wistar rats were received from (Charles River, Frederick, MD). Sixteen of the rats were implanted with Physio Tel Multiplus transmitters placing the biopotential wires in a lead II ECG configuration and the pressure cannula in the descending aorta. The other four rats were naïve and were intended for drug concentration measurements. After two weeks of recovery, the sixteen rats were randomly assigned to two groups. One group of eight rats was targeted for a study using the ABST system whereas the second group of eight was studied using the traditional DSI home cage configuration. For ABST studies, animals were further implanted with carotid artery cannulas for automated blood sampling. For the traditional DSI home cage configuration the additional and separate group of four non-telemetered 'satellite' animals were cannulated via carotid artery and used for blood sampling to determine comparable drug concentration data. The satellite rats were used in the study in order to minimize handling of the traditional DSI telemetry group which would otherwise alter physiological recordings. For all cannulated animals, a 1:1 heparin-glycerol solution was injected into the carotid cannula to maintain the patency before being exteriorized between the scapulae. Four days post-surgery, animals intended for study with the ABST were acclimated to the system (Figure 4), while the rats destined for traditional telemetry remained in their home cages. On the day of the experiment, a 60-minute baseline period was recorded from both groups before dosing with baclofen (10 mg/kg, p.o.) or vehicle. Mean arterial pressure (MAP) was recorded from each of the rats for up to 12 hours following dosing. At the end of recording all ABST animals were returned to their home cages. Seventy-two hours after the first dose, a treatment crossover was performed within each system such that each rat received the opposite treatment (baclofen or vehicle). At the end of the experiment each rat had received both treatments and therefore served, as its own control within a given experimental paradigm. Blood samples collected on the ABST and from the satellite rats were deposited in tubes containing 10 μl EDTA and maintained at 4°C until the end of the experiments. Blood samples were subsequently centrifuged at 1500 rpm for 30 minutes; plasma samples were extracted and stored at -80°C until analyzed.

The responses in both groups of animals were compared using repeat measures ANOVA with a Bonferroni post hoc analysis set at p < 0.05. MAP data are presented as mean ± SEM whereas drug concentrations are represented as mean ± SD.

### Determination of the physiological concentrations of baclofen in rat plasma following an acute exposure

**Figure 5:**
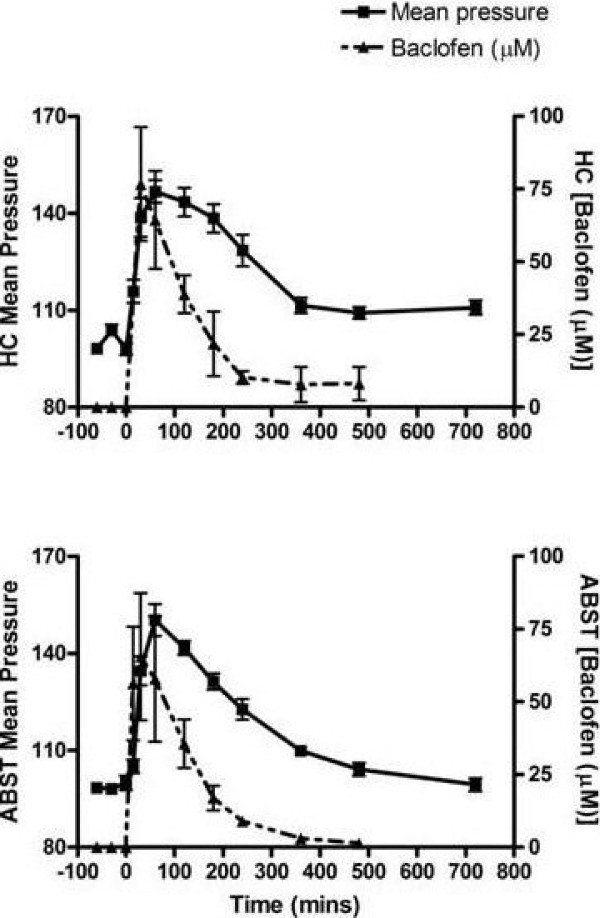
**Mean Arterial Pressure Response to Baclofen**. The effect of a single dose of baclofen (10 mg/Kg p.o.) on mean arterial pressure (MAP). Figure 5a illustrates changes in MAP (%) from control on the left Y-axis and the physiological concentration of baclofen (μM) on the right Y-axis. The responses recorded on the traditional telemetry system are shown on the bottom while responses on the Automated Blood Sampling and Telemetry (ABST) system are shown on the right.

The Drug Metabolism and Pharmacokinetic (DMPK) Department at AstraZeneca Pharmaceuticals determined levels of baclofen in the rat EDTA plasma (see Figure 5). Briefly, samples were extracted using 95% acetonitrile/1% Formic acid solvent and spiked with the appropriate internal standard (137 nM labetalol). Samples and standards were injected into an Agilent Zorbax SB-C18 300 column (30 × 2.1 mm, 3.5 μm) using 95% acetonitrile/1% formic acid mobile phase. Baclofen and its internal standard were detected using a Micromass Ultima Triple Quadrupole LC-MS/MS, with an ESI interface.

## Results

### Mean arterial pressure (MAP) effects of baclofen

The MAP responses to orally administered doses of baclofen were qualitatively and quantitatively similar in both systems. Baclofen produced comparable increases in MAP (ABST vs. traditional DSI telemetry rats (mm Hg): 150 ± 5 vs.147 ± 4, p = NS, see Figure 5). In addition, the changes observed in MAP occurred in response to similar peak concentrations of baclofen (ABST vs. satellite rats (μM): 69.6 ± 23.8 vs. 76.6 ± 19.5, p = NS, see Figure 5).

## Discussion

We describe a unique automated blood sampling and telemetry (ABST) system. Combination of the equipment was achieved by designing and optimizing a circular antenna to facilitate detection of radio telemetric recordings that would not interfere with synchronized Culex^® ^computer-automated blood sampling. The antenna design allows optimal detection of high fidelity radio telemetry signals within the cage while reducing noise from adjacent radio telemetry equipment. There are several advantages to using the ABST system as an experimental platform.

In order to exemplify one advantage of the ABST system over traditional approaches, mean arterial pressure (MAP) responses to baclofen, an analogue of gamma amino butyric acid that acts as a GABA_B _receptor agonist were measured. At physiological concentrations greater than 500 nM baclofen causes a marked and prolonged increase in blood pressure [11,12]. In this study, similar baclofen concentrations and MAP responses were obtained using both ABST and traditional approaches. With the ABST system baclofen concentration profile and MAP responses were matched in the same animal as opposed to traditional approaches that required the use of additional satellite rats for determination of baclofen concentration. Using the ABST system multiple parameters can be measured in the same study and therefore improve data comparisons by removing potential confounding covariates that can exist between studies.

**Figure 6:**
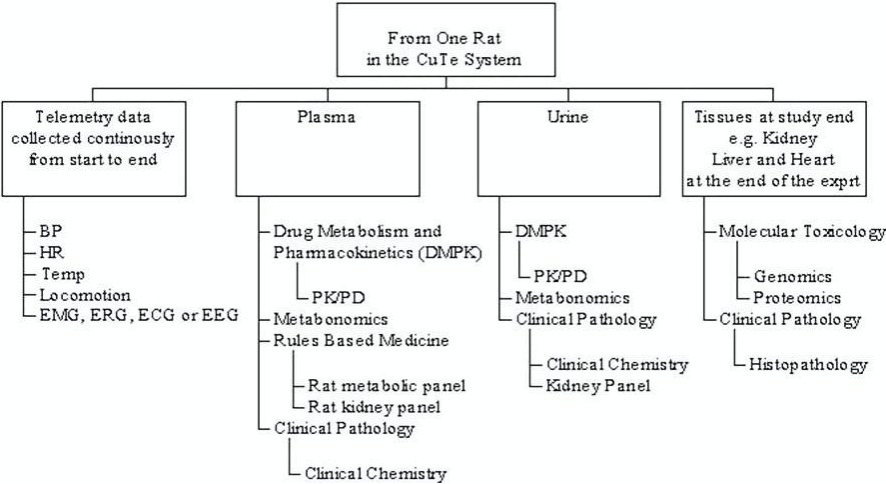
**Illustration of variables that can be measured in one animal using the ABST system**. Multiple physiological variables including blood pressure (BP), heart rate (HR), temperature (Temp), locomotion, activity and various biopotential leads e.g. electrocardiographs (ECG), electroencephalographs (EEG) electromyographs (EMG), or electroretinographs (ERG) can be measured in one rat concomitantly with plasma and metabolic waste. Plasma and urine samples can be used for determination of drug concentration time course and characterization of multiple biochemical endpoints such as clinical pathology, urinary renal biomarkers as well as metabonomics. If the study requires histopathology, genomic and proteomic endpoints, target organ samples are available at the end of the "live phase".

Using whole animal models to measure diverse organ functions *in vivo *can reduce the need for multiple and separate studies [13]. As shown in Figure 6, many variables can be measured using the ABST system. Advantages of measuring multiple organ variables in ABST include: (a) the significant reduction of total combined experiment preparation time, manpower and other resources i.e. consumable supplies; (b) reduction of the quantity of test compound required; (c) identification of outlier animals that are unresponsive or hyper-responsive to test compounds because exposures are determined in the same animal; (d) a unique opportunity to examine individual subject variability against a parameter of interest, and; (e) reducing the total number of animals used for study.

Perhaps the most useful advantage is the potential for the ABST system to enable multivariate and pair-wise comparisons of data. For example, we have previously shown concomitant recording of physiological effects including hemodynamic variables, heart rate, body temperature, locomotion and EEG with biochemical endpoints such as renal biomarkers measured from urine and blood in the same animal [13]. Paired comparisons can also facilitate direct analyses of temporal relationships existing between variables. An additional and direct benefit of this approach is the ability to sequentially test drug exposures ranging from therapeutic to toxicologically-relevant levels in a single *in vivo *study in order to provide more definitive margin-of-safety estimates for a given therapeutic target.

## Conclusions

A novel circular antenna compatible with DSI radio telemetry equipment was engineered to enable *in vivo *physiologic recordings simultaneous with automated blood sampling using the BASi Culex^® ^device. The integration of previously existing stand-alone systems allows for improved data quality while significantly reducing animal use. The system also provides an opportunity to evaluate temporal relationships between multiple parameters and to investigate anomalous outlier events because drug concentrations, physiological and biochemical measures for each animal are available for comparisons.

## List of abbreviations

%: Per cent; ABS: Automated blood sampler; ABST: Automated Blood Sampling and Telemetry; ANOVA: Analysis of Variance; BASi: Bioanalytical Systems incorporated; Cmax: Maximum concentration; DMPK: Department of metabolism and pharmacokinetics; DSI: Data Sciences International; ECG: Electrocardiogram; EDTA: Ethylenediaminetetraacetic acid; EEG: Electroencephalogram; ESI: Electrospray ionization; GABA_B_: Gamma amino butyric acid; LC-MS/MS: Liquid chromatography/mass spectrometry; LCR meter: Inductance (L), Capacitance (C), and Resistance (R)) meter; MAP: Mean Arterial Pressure; MD: Maryland; Mg/Kg: Milligrams per Kilogram; mm Hg: Millimeters of mercury; MN: Minnesota; nM: Nanomolar; NS: Not significant; °C: Degrees centigrade; p: Probability value; p.o.: Per os; RPM: Rotations per minute; SD: Standard deviation; SEM: Standard error of the mean; μM: Micromolar

## Competing interests

The authors declare that they have no competing interests.

## Authors' contributions

DCL re-engineered the DSI telemetry and helped to draft the manuscript. DJL and RAB conceived of the study, and participated in its design and coordination and helped to draft the manuscript. HWK designed some of the experimental work and helped draft the manuscript. All authors read and approved the final manuscript.
